# Supplementary material for: Advancing Cholangiocarcinoma Care: Insights and Innovations in T Cell Therapy
Source: Cancers (Basel). 2024 Sep 23;16(18):3232. doi: 10.3390/cancers16183232 (PMC11429565; doi:10.3390/cancers16183232)
Supplement: Supplementary file 1 [file cancers-16-03232-s001.zip › cancers-3194587-supplementary.pdf]

Table S1-Summary of research studies evaluating the Immune Cell Dynamics and the prognostic, therapeutic relevance of specific immune cell subsets in CCA patients

**Authors**	**Immune cell subset evaluated**	**Summary of the study findings**	**Ref.**
Lin J et al (2022)	Functional subgroups	Three immune subgroups in CCA designated as IG1 (immune-suppressive, 25.1%), IG2 (immune-exclusion, 42.7%), and IG3 (immune-activated, 32.2%). The three groups differ in their clinical and immunogenomic traits highlighting the importance of personalized immunotherapy	[1]
Lin Y et al (2022)	Infiltration subgroups	Using multiple sequencing modalities, the intratumoral heterogeneity of immune infiltration was characterized and ICC were classified into sparsely, heterogeneously, and highly infiltrated subgroups with distinct immune evasion mechanisms	[2]
Kim et al (2021)	CD4 T cells	The active infiltration of T cell subsets with higher levels of LAG3 and TIM3 expression is seen in the tumor margin. The density of tumor margin-infiltrating FoxP3- CD4+ helper T cells is associated with better clinical outcomes.	[3]
Asahi Y et al (2021)	CD8 T cells	CD8+ T cell infiltration correlates with the HLA Class I expression on the tumor cells. Patients with increased CD8+ T cell infiltration in the tumor margins had better survival.	[4]
Tsukagoshi M et al (2016)	NK cells	Concurrent high expression of multiple NKG2D ligands revealed significantly better overall and disease-free survival in CCA	[5]
Zhou G et al (2020)	NK T cells	Decrease in the proportions of NKT cells in the tumor and reduced perforin secretion from the NKT cells in the tumor tissue compared to adjacent tumor free liver tissue.	[6]
Zhang T et al (2022)	Gamma Delta T cells	Adoptive transfer of allogeneic γδ cells was safe, with encouraging clinical efficacy against HCC and CCA	[7]
Martin-Sierra C et al (2018)	Dendritic cells (DC)	Decrease in the frequency and the absolute numbers of circulating monocytes and mDCs in CCA patients. The mDCs from CCA patients were dysfunctional with reduced TNFα production compared to healthy controls	[8]
Hu Z et al (2020)	pDC	Larger numbers of peri-tumoral pDCs and increased Foxp3+ regulatory T cell infiltration was associated with larger tumors, advanced staging with metastasis and poor overall survival.	[9]
Konishi D et al (2022)	T Regs	Increased FoxP3+/CD8+ ratio was an important marker for poor survival and showed a positive correlation with lymph node metastasis	[10]
Xu XD et al (2016)	MDSC	CD14+/CD11b+/HLA-DR- MDSCs and serum GM-CSF levels were increased in patients with pancreatic cancer and CCA and the levels correlated with clinical cancer stage.	[11]
Thanee M et al (2015)	TAM	CCA induces polarization of macrophages to M2 phenotype (TAM) which is associated with extrahepatic metastasis and poor survival.	[12]
Kitano Y et al (2018)	TAN	TANs showed a positive correlation with TRegs and negative correlation with CD8+ T cells and was associated with poor survival and distant metastasis.	[13]

Table S2-overview of research studies focused on CCA, detailing the diversity of biomarkers evaluated, such as cytokines, immune checkpoints, and cell surface proteins

**Author**	***Markers***	***Diagnoses***	**Ref**
Joon-Yong Chung	PTEN (Phosphatase and Tensin Homolog), p-AKT (Phosphorylated AKT), p-mTOR (Phosphorylated mammalian Target of Rapamycin)	ICC	[14]
Yu-HuaHuang	CD20, CD3, CD68, CD8, FoxP3, cytotoxic T-lymphocyte-associated protein 4 (CTLA-4), HLA-DR and CD163, TMA , (PD-L1, PD-1	ICC	[15]
M B Sturm	EGFR, claudin-1, ErbB2 ,	ICC	[16]
Lingyu Tian	PD-1/PD-L1,CD8,CD68	ICC	[17]
Hyung-Don Kim	EBNA1, LMP1, EBNA2, PD-1, PD-L1, CD3+, CD8+, CD20+	ICC	[3]
Guang-Yu Ding	CD4, Bcl6, Foxp3	iCC	[18]
Hyung-Don Kim	CD69, CD103, CD8, PD-L1, Ki-67, HLA-DR, CD38, and CD39	ICC	[19]
Tao Xia	CD8, Granzyme B, PD-1, EOMES, PD-L1, CD68, Foxp3	ICC, HC, DCC	[20]
Liwei Chen	CD31, α-SMA (Alpha-Smooth Muscle Actin), and Angiopoietin-1 (Ang-1).	ICC	[21]
Naoki Yagi	Glypican-3 (GPC3), Cytokeratin 7 (CK7), CD3, Programmed Death-Ligand 1 (PD-L1), CD8, CD163, and CD204	cHCC-CCA	[22]
Chunbin Zhu	CD15, CD206, PD-L1, CD3, CD4, CD8, FoxP3, CD68, CD20, Granzyme B, Perforin, CD45, CD56, CD11b, CD11c, CD163, CD57, HLA-DR, Ki-67, TIA-1, PD-1, CTLA-4, LAG-3, TIM-3, MHC Class I, MHC Class II, FAP, VISTA, ICOS, B7-H3, B7-H4, GITR, OX40, IDO, TGF-β, IFN-γ, TNF-α	ICC	[23]
Jing Han Hong	PD-L1, CS56, PD-L2, CD68, CD8, FOXP3	ICC	[24]
Zixue Xuan	D4, CD146, S100P, SPP1, MS4A1	ICC	[25]

Table S3- Ongoing Immunotherapy Trials in Bile Duct Cancer (CCA) - Exploring Novel Treatment Approaches

**Sponnser**	**Title**	**Drug**	**Target**	**Trial registration**
Charbel Elias	A phase II single arm study of Nivolumab with stereotactic Ablative radiation Therapy after induction chemotherapy in CCA (NATCHO)	Nivolumab	PD-1	NCT04648319
University of Alabama at Birmingham	An Open-label Window of Opportunity Trial to Evaluate the Activity of Durvalumab (MEDI4736) and Tremelimumab With Platinum-based Chemotherapy (Gemcitabine and Cisplatin) in Intrahepatic CCA (ICC)	Durvalumab Tremelimumab	PD-L1 (CD274) CTLA-4	NCT04989218
Zhejiang Cancer Hospital	Prospective Observational Study on Prediction of Response to First-line Immunotherapy in Patients With Biliary Tract Tumors			
Fudan University	A Phase II Study of Lenvatinib Plus Sintilimab in Patients With **Immune Checkpoint Inhibitor** Previously Treated Advanced Liver Cancer	Sintilimab	PD-1	NCT05010681
Second Affiliated Hospital, School of Medicine, Zhejiang University	A Phase Ⅱ, Open Label, Single-center Study of Lenvatinib and Tirelizumab Combined With Gemcitabine and Cisplatin (GPLET) in the Treatment of Advanced **CCA**	Tislelizumab	PD-1	NCT05532059
Second Affiliated Hospital, School of Medicine, Zhejiang University	A Randomized, Double-blind, Multicenter Study of Lenvatinib, Temalizumab Combined With Gemcitabine and Cisplatin (GPLET) in the Treatment of Advanced **CCA**	Tislelizumab	PD-1	NCT05823311
Zhejiang Cancer Hospital	Tislelizumab Combined With GEMOX (GOT) Applied as Neoadjuvant Regimen for Patients of ResectableIntrahepatic **CCA** With High-risk Factors of Recurrence: a Single Arm, Single Center, Prospective, Explorative Clinical Trail.	Tislelizumab	PD-1	NCT05557578
University of Florida	Atezolizumab Plus Tivozanib in Immunologically Cold Tumor Types	Atezolizumab	PD-L1	NCT05000294
Institut für Klinische Krebsforschung IKF GmbH at Krankenhaus Nordwest	A Phase II Single-arm, Open-label Study of Atezolizumab and Derazantinib for Patients With Advanced Intrahepatic CCA With FGFR2	Atezolizumab	PD-L1	NCT05174650

Halozyme Therapeutics	A Phase 1B, Randomized, Open-Label Study of PEGylated Recombinant Human Hyaluronidase (PEGPH20) in Combination With Cisplatin Plus Gemcitabine and PEGPH20 in Combination With Atezolizumab and Cisplatin Plus Gemcitabine Compared With Cisplatin Plus Gemcitabine in Subjects With Previously Untreated, Unresectable, Locally Advanced, or Metastatic Intrahepatic and Extrahepatic CCA and Gallbladder Adenocarcinoma	Atezolizumab	PD-L1	NCT03267940
Aiwu Ruth He, MD	A Phase II Multi-center Study Evaluating Combination Immunotherapy for Advanced CCA With Pembrolizumab and Sylatron (Peginterferon Alfa-2b) HCRN:GI16-263	Pembrolizumab	PD-1	NCT02982720
M.D. Anderson Cancer Center	A Single-Arm Study of Pembrolizumab With Gemcitabine and Cisplatin as Perioperative Therapy for Potentially Resectable Intrahepatic CCA	Pembrolizumab	PD-1	NCT05967182
Nataliya Uboha	Study of Gemcitabine, Cisplatin, Quemliclustat (AB680) and Zimberelimab (AB122) During First Line Treatment of Advanced Biliary Tract Cancers (BTC).	Zimberelimab	PD-1	NCT06048133
National Cancer Institute (NCI)	A Randomized Phase II Trial Evaluating Chemotherapy Plus Atezolizumab vs Chemotherapy Plus Bevacizumab and Atezolizumab in Advanced Combined Hepatocellular Carcinoma-CCA	Atezolizumab	PD-L1	NCT05211323
Jason J. Luke, MD	Evaluating Length of Treatment With PD-1/PD-L1 Inhibitor in Advanced Solid Tumors	Pembrolizumab<br>Nivolumab<br>Atezolizumab<br>Ipilimumab<br>Cemiplimab	PD-1<br>PD-1<br>PD-L1<br>CTLA-4<br>PD-1	NCT04157985
NGM Biopharmaceuticals, Inc	A Phase 1 Dose Escalation/Dose Finding Study of NGM831 as Monotherapy and in Combination With Pembrolizumab or Pembrolizumab and NGM438 in Advanced or Metastatic Solid Tumors	NGM831	ILT3	NCT05215574
NGM Biopharmaceuticals, Inc	A Phase 1/1b Dose Escalation/Expansion Study of NGM438 as Monotherapy and in Combination With Pembrolizumab in Advanced or Metastatic Solid Tumors	NGM438	LAIR1	NCT05311618
NGM Biopharmaceuticals, Inc	A Phase 1/2 Dose Escalation/Expansion Study of NGM707 as Monotherapy and in Combination With Pembrolizumab in Advanced or Metastatic Solid Tumor Malignancies	NGM707	ILT2 and ILT4	NCT04913337

Xencor, Inc.	A Phase 1 Multiple-Dose Study to Evaluate the Safety and Tolerability of XmAb®22841 Monotherapy and in Combination With Pembrolizumab in Subjects With Selected Advanced Solid Tumors (DUET-4)	XmAb®22841	CTLA-4 x LAG-3	NCT03849469
Merck Sharp & Dohme LLC	Study of Pembrolizumab (MK-3475) in Participants With Advanced Solid Tumors (MK-3475-158/KEYNOTE-158)	Pembrolizumab	PD-1	NCT02628067
National Cancer Institute (NCI)	A Phase 2 Study of Pembrolizumab, a Monoclonal Antibody Against PD-1, in Combination With Capecitabine and Oxaliplatin (CAPOX) in Subjects With Advanced Biliary Tract Carcinoma (BTC)	Pembrolizumab	PD-1	NCT03111732
Celldex Therapeutics	A Study of CDX-1140 (CD40) as Monotherapy or in Combination in Patients With Advanced Malignancies	CDX-1140 (CD40)	CD40	NCT03329950
SOLTI Breast Cancer Research Group	Efficacy of Tislelizumab and Spartalizumab Across Multiple Cancer-types in Patients With PD1-high mRNA Expressing Tumors Defined by a Single and Pre-specified Cutoff	Tislelizumab<br>Spartalizumab	PD-1<br>PD-1 (CD279)	NCT04802876

Table S4- Research studies on the prognostic significance of tumor-infiltrating lymphocytes (TILs) and related immune markers in CCA (CCA), categorized by pre-clinical, animal model, and clinical studies.

**Author**	**Date**	**Summary**	**Ref.**
**Cheng Z et al**	2021	NOC and LAIR2 were biomarkers for immune infiltration evaluation in CCA. PNOC, expressed by B cells, could predict better survival of patients, while LAIR2 was a potential marker for exhaustive T cell populations, correlating with worse survival of patients.	[26]
**Yugawa K et al**	2021	A decreased intratumoral MVD might predict ICC patient outcomes. Tumor microvessels might be associated with ICC progression, possibly by altering TIL recruitment.	[27]
**Qiang Z et al**	2022	For the prediction of the PFS and OS of patients with ICC after complete resection, stromal TILs play an important role.	[28]

**Alvisi G et al**	2022	Abundant infiltration of hyperactivated CD4+ Tregs in ICC tumors along with reduced CD8+ T-cell effector functions was observed. Interfering with hyperactivated Tregs should be explored as an approach to enhance antitumor immunity in ICC.	[29]
**Sinclair White B et al**	2023	*FGFR2* fusion-reactive TILs can be isolated from some patients with metastatic ICC, and thus provides a rationale for future exploration of T cell-based therapy targeting *FGFR2* fusions in patients with cancer.	[30]
**Ma W et al**	2023	The tumor microbiome is related to CD8+ T lymphocyte infiltration in patients with resected BTCs. The relationship between tumor Clostridia and high infiltration of CD8+ T lymphocytes might reflect decreased recruitment of myeloid-derived suppressor cells via the PI3K-CCL2-CCR2 axis	[31]
**Ye Y et al**	2009	B7-H1/PD-1 pathway may be linked to malignant potential of ICC and contribute to tumor immune evasion by promoting CD8+ TILs apoptosis. Thus, this pathway may indeed be a potential therapeutic target in the treatment of this disease.	[32]
Zhao X et al	2016	Aberrant expression of B7-H4 was correlated with poorer prognosis and suppressed CD8+ in CCA.	[33]
**Jin Lim Y et al**	2017	This study demonstrates a potential prognostic relevance of CTLA-4 expression in EHBD cancer.	[34]
Kim R et **al**	2018	Neither CD8+TIL nor PD-L1 expression on cancer cells correlated significantly with OS. These results add to the understanding of the clinical features associated with CD8 TILs and PD-L1 expression in extrahepatic CCA, and they support the potential rationale of using PD-1 blockade immunotherapy in CCA.	[35]
**Kitano Y et al**	2018	Tumour infiltrating inflammatory and immune cells may play a pivotal role in ECC progression and a high-risk signature predicted poor prognosis in ECC patients.	[36]
**Vigano L et al**	2019	TILs are associated with prognosis of ICC patients after complete surgery. CD3+ and CD8+ infiltrate is associated with higher survival and lower recurrence risk, while Foxp3+ infiltrate is associated with worse prognosis. CD3+ infiltrate allows refining prediction of prognosis in early tumors.	[37]
Yu F et al	2019	PD-L1 expression in association with intra-tumoral TILs infiltration and HLA class I expression in 32.3% of the ECC reflects an active immune microenvironment potentially responsive to PD-1/PD-L1 inhibitors. In addition, the combination of macrophage-targeting agents may provide therapeutic synergy for future immunotherapy.	[38]
**Handrup Kverneland A et al**	2021	High success rates of TIL expansion were demonstrated across multiple solid cancers. TIL ACTs were found feasible, independent of previous therapy. Tumor regressions after ACT combined with CPIs were demonstrated in several cancer types supported by in vitro antitumor reactivity of the TILs.	[39]
Miyazaki K et al	2021	Stromal TILs, especially in the marginal area, might demonstrate prognostic impact in patients with IHCC. Moreover, the ADC values from MRI may predict TILs in IHCC tumor tissue.	[40]
**Miyazaki K et al**	2021	The preoperative LCR may predict the postsurgical prognosis of patients with IHCC and reflect the $CD8^+$ TILs.	[41]
**Kim H-D et al**	2021	The tumor margin is the major site for the active infiltration of T cell subsets with higher levels of LAG3 and TIM3 expression in BTC. The density of tumor margin-infiltrating FoxP3- CD4+ helper T cells may be associated with clinical outcomes in BTC patients treated with gemcitabine plus cisplatin.	[42]
**Intarawichian et al**	2022	TILs could be a prognostic factor for predicting survival and for clustering patients with dCCA to improve prognostication capability.	[43]
Lin Z et al	2022	LMR played as an independent factor for predicting the survival in patients with HCCA after R0 radical resection. A high LMR was associated with an accumulation of $CD3^+$ T cells in HCCA.	[44]

**Lan C et al**	2022	Cellular senescence, represented by CAV1 levels, may be a marker of CAFs and a prognostic indicator of ICC through Foxp3+ TIL regulation. CAV1 expression in CAFs can be a therapeutic target for ICC.	[45]
**Byeon S et al**	2023	Immune cell PD-L1-positivity and the administration of adjuvant chemotherapy may indicate the favorable survival of patients with surgically-resected biliary tract cancers, specifically, in the tumoral PD-L1$^{(-)}$ or tumor leptin$^{(-)}$ subgroups and extrahepatic CCA. PD-L1- or leptin-targeted therapy combined with conventional chemotherapy may benefit the tumoral PD-L1^{+} or leptin^{+} subgroups.	[46]

1. Lin, J., et al., *Multimodule characterization of immune subgroups in intrahepatic cholangiocarcinoma reveals distinct therapeutic vulnerabilities.* J Immunother Cancer, 2022. **10**(7).
2. Lin, Y., et al., *Geospatial Immune Heterogeneity Reflects the Diverse Tumor-Immune Interactions in Intrahepatic Cholangiocarcinoma.* Cancer Discov, 2022. **12**(10): p. 2350-2371.
3. Kim, H.D., et al., *Spatial Distribution and Prognostic Implications of Tumor-Infiltrating FoxP3- CD4+ T Cells in Biliary Tract Cancer.* Cancer Res Treat, 2021. **53**(1): p. 162-171.
4. Asahi, Y., et al., *Prognostic impact of CD8+ T cell distribution and its association with the HLA class I expression in intrahepatic cholangiocarcinoma.* Surg Today, 2020. **50**(8): p. 931-940.
5. Tsukagoshi, M., et al., *Overexpression of natural killer group 2 member D ligands predicts favorable prognosis in cholangiocarcinoma.* Cancer Sci, 2016. **107**(2): p. 116-22.
6. Zhou, G., et al., *Reduction of immunosuppressive tumor microenvironment in cholangiocarcinoma by ex vivo targeting immune checkpoint molecules.* J Hepatol, 2019. **71**(4): p. 753-762.
7. Zhang, T., et al., *Clinical Safety and Efficacy of Locoregional Therapy Combined with Adoptive Transfer of Allogeneic gammadelta T Cells for Advanced Hepatocellular Carcinoma and Intrahepatic Cholangiocarcinoma.* J Vasc Interv Radiol, 2022. **33**(1): p. 19-27 e3.
8. Martin-Sierra, C., et al., *Functional Impairment of Circulating FcepsilonRI(+) Monocytes and Myeloid Dendritic Cells in Hepatocellular Carcinoma and Cholangiocarcinoma Patients.* Cytometry B Clin Cytom, 2019. **96**(6): p. 490-495.
9. Hu, Z.Q., et al., *Peritumoral plasmacytoid dendritic cells predict a poor prognosis for intrahepatic cholangiocarcinoma after curative resection.* Cancer Cell Int, 2020. **20**(1): p. 582.
10. Konishi, D., et al., *Regulatory T cells induce a suppressive immune milieu and promote lymph node metastasis in intrahepatic cholangiocarcinoma.* Br J Cancer, 2022. **127**(4): p. 757-765.
11. Xu, X.D., et al., *Circulating myeloid-derived suppressor cells in patients with pancreatic cancer.* Hepatobiliary Pancreat Dis Int, 2016. **15**(1): p. 99-105.
12. Thanee, M., et al., *Quantitative changes in tumor-associated M2 macrophages characterize cholangiocarcinoma and their association with metastasis.* Asian Pac J Cancer Prev, 2015. **16**(7): p. 3043-50.
13. Kitano, Y., et al., *Tumour-infiltrating inflammatory and immune cells in patients with extrahepatic cholangiocarcinoma.* Br J Cancer, 2018. **118**(2): p. 171-180.
14. Chung, J.-Y., et al., *The expression of phospho-AKT, phospho-mTOR, and PTEN in extrahepatic cholangiocarcinoma.* Clinical cancer research, 2009. **15**(2): p. 660-667.

15. Huang, Y.-H., et al., *Clinicopathologic features, tumor immune microenvironment and genomic landscape of Epstein-Barr virus-associated intrahepatic cholangiocarcinoma.* Journal of hepatology, 2021. **74**(4): p. 838-849.
16. Sturm, M., et al., *Multiplexed imaging strategy to distinguish indeterminant biliary strictures: An ex vivo study.* World journal of gastroenterology, hepatology and endoscopy, 2020. **3**(3).
17. Tian, L., et al., *PD-1/PD-L1 expression profiles within intrahepatic cholangiocarcinoma predict clinical outcome.* World journal of surgical oncology, 2020. **18**(1): p. 1-11.
18. Ding, G.-Y., et al., *Distribution and density of tertiary lymphoid structures predict clinical outcome in intrahepatic cholangiocarcinoma.* Journal of Hepatology, 2022. **76**(3): p. 608-618.
19. Kim, H.D., et al., *Implication of CD69+ CD103+ tissue-resident-like CD8+ T cells as a potential immunotherapeutic target for cholangiocarcinoma.* Liver International, 2021. **41**(4): p. 764-776.
20. Xia, T., et al., *Immune cell atlas of cholangiocarcinomas reveals distinct tumor microenvironments and associated prognoses.* Journal of Hematology & Oncology, 2022. **15**(1): p. 1-20.
21. Chen, L., et al., *The impact of decreased expression of SVEP1 on abnormal neovascularization and poor prognosis in patients with intrahepatic cholangiocarcinoma.* Frontiers in Genetics, 2023. **13**: p. 1127753.
22. Yagi, N., et al., *Component with abundant immune-related cells in combined hepatocellular cholangiocarcinoma identified by cluster analysis.* Cancer Science, 2022. **113**(5): p. 1564-1574.
23. Zhu, C., et al., *Spatial immunophenotypes predict clinical outcome in intrahepatic cholangiocarcinoma.* JHEP Reports, 2023: p. 100762.
24. Hong, J.H., et al., *Integrative multiomics enhancer activity profiling identifies therapeutic vulnerabilities in cholangiocarcinoma of different etiologies.* Gut, 2023.
25. Xuan, Z., et al., *Novel cell subtypes of SPP1+ S100P+, MS4A1-SPP1+ S100P+ were key subpopulations in intrahepatic cholangiocarcinoma.* Biochimica et Biophysica Acta (BBA)-General Subjects, 2023. **1867**(9): p. 130420.
26. Chen, Z., et al., *PNOC expressed by B cells in cholangiocarcinoma was survival related and LAIR2 could be a T cell exhaustion biomarker in tumor microenvironment: characterization of immune microenvironment combining single-cell and bulk sequencing technology.* Frontiers in Immunology, 2021. **12**: p. 647209.
27. Yugawa, K., et al., *Prognostic impact of tumor microvessels in intrahepatic cholangiocarcinoma: association with tumor-infiltrating lymphocytes.* Modern Pathology, 2021. **34**(4): p. 798-807.
28. Qiang, Z., et al., *The prognostic value of stromal tumor-infiltrating lymphocytes in intrahepatic cholangiocarcinoma: a population-based study.* Scandinavian Journal of Gastroenterology, 2022. **57**(8): p. 965-971.
29. Alvisi, G., et al., *Multimodal single-cell profiling of intrahepatic cholangiocarcinoma defines hyperactivated Tregs as a potential therapeutic target.* Journal of Hepatology, 2022. **77**(5): p. 1359-1372.
30. White, B.S., et al., *Specific recognition of an FGFR2 fusion by tumor infiltrating lymphocytes from a patient with metastatic cholangiocarcinoma.* Journal for Immunotherapy of Cancer, 2023. **11**(4).
31. Ma, W.-J., et al., *PI3K-CCL2-CCR2-MDSCs axis: A potential pathway for tumor Clostridia-promoted CD 8+ T lymphocyte infiltration in bile tract cancers.* Neoplasia, 2023. **43**: p. 100920.
32. Ye, Y., et al., *Interaction of B7-H1 on intrahepatic cholangiocarcinoma cells with PD-1 on tumor-infiltrating T cells as a mechanism of immune evasion.* Journal of surgical oncology, 2009. **100**(6): p. 500-504.
33. Zhao, X., et al., *Aberrant expression of B7-H4 correlates with poor prognosis and suppresses tumor-infiltration of CD8+ T lymphocytes in human cholangiocarcinoma.* Oncology Reports, 2016. **36**(1): p. 419-427.

34. Lim, Y.J., et al., *Clinical implications of cytotoxic T lymphocyte antigen-4 expression on tumor cells and tumor-infiltrating lymphocytes in extrahepatic bile duct cancer patients undergoing surgery plus adjuvant chemoradiotherapy.* Targeted oncology, 2017. **12**: p. 211-218.
35. Kim, R., et al., *Prognostic value of CD8CD45RO tumor infiltrating lymphocytes in patients with extrahepatic cholangiocarcinoma.* Oncotarget, 2018. **9**(34): p. 23366.
36. Kitano, Y., et al., *Tumour-infiltrating inflammatory and immune cells in patients with extrahepatic cholangiocarcinoma.* British journal of cancer, 2018. **118**(2): p. 171-180.
37. Vigano, L., et al., *Tumor-infiltrating lymphocytes and macrophages in intrahepatic cholangiocellular carcinoma. Impact on prognosis after complete surgery.* Journal of Gastrointestinal Surgery, 2019. **23**: p. 2216-2224.
38. Yu, F., et al., *Programmed death ligand-1, tumor infiltrating lymphocytes and HLA expression in Chinese extrahepatic cholangiocarcinoma patients: possible immunotherapy implications.* Bioscience trends, 2019. **13**(1): p. 58-69.
39. Kverneland, A.H., et al., *Adoptive cell therapy with tumor-infiltrating lymphocytes supported by checkpoint inhibition across multiple solid cancer types.* Journal for immunotherapy of cancer, 2021. **9**(10).
40. Miyazaki, K., et al., *Stromal tumor-infiltrating lymphocytes level as a prognostic factor for resected intrahepatic cholangiocarcinoma and its prediction by apparent diffusion coefficient.* International Journal of Clinical Oncology, 2021. **26**: p. 2265-2274.
41. Miyazaki, K., et al., *Preoperative lymphocyte/C-reactive protein ratio and its correlation with CD8+ tumor-infiltrating lymphocytes as a predictor of prognosis after resection of intrahepatic cholangiocarcinoma.* Surgery Today, 2021. **51**(12): p. 1985-1995.
42. Kim, H.-D., et al., *Spatial distribution and prognostic implications of tumor-infiltrating FoxP3-CD4+ T cells in biliary tract cancer.* Cancer Res Treat, 2021. **53**(1): p. 162-171.
43. Intarawichian, P., et al., *Prognostic significance of tumor-infiltrating lymphocytes in predicting outcome of distal cholangiocarcinoma in Thailand.* Frontiers in Oncology, 2022. **12**: p. 1004220.
44. Lin, Z.-q., et al., *Prognostic significance of NLR, PLR, LMR and tumor infiltrating T lymphocytes in patients undergoing surgical resection for hilar cholangiocarcinoma.* Frontiers in Oncology, 2022. **12**: p. 908907.
45. Lan, C., et al., *Cancer-associated fibroblast senescence and its relation with tumour-infiltrating lymphocytes and PD-L1 expressions in intrahepatic cholangiocarcinoma.* British journal of cancer, 2022. **126**(2): p. 219-227.
46. Byeon, S.j., et al., *Prognostic roles of leptin-signaling proteins, PD-L1, and tumor-infiltrating lymphocytes in surgically-resected biliary tract cancers.* Journal of Surgical Oncology, 2023. **127**(4): p. 587-597.
